# Post-Mortem Characterization of Cerium Speciation in Molten Calcium Chloride

**DOI:** 10.1021/acs.inorgchem.6c02415

**Published:** 2026-09-09

**Authors:** Sheridon N. Kelly, Alfred Amon, Maryline G. Ferrier, Karla A. Erickson, L. Eddy Banks, Marisa J. Monreal, Bradley C. Childs

**Affiliations:** † Physical and Life Sciences Directorate, 4578 Lawrence Livermore National Laboratory, Livermore, California 94550, United States; ‡ Chemistry Division, 5112 Los Alamos National Laboratory, Los Alamos, New Mexico 87545, United States

## Abstract

Advancing radiochemistry and nuclear materials science requires understanding actinide interactions with molten salts, which are used in next-generation nuclear reactors and for processing of actinides to recover useful fissile material. Understanding chemical interactions of actinides with molten salts has been limited by challenges in developing spectroscopic and X-ray techniques that are compatible with the high temperatures necessary to study molten salts. In this work, interactions of CeO_2_ (serving as a nonradioactive surrogate for uranium and plutonium) with CaCl_2_ are characterized following heating. Scanning electron microscopy indicates CeO_2_ morphological changes from small (<1 μm) particles to 3–5 μm sheets. Powder X-ray diffraction and infrared and Raman spectroscopies show the formation of CeOCl at higher (850–1050 °C) temperatures. In the absence of a chemical reducing agent, it was found that a high-temperature, low-oxygen environment is the key to the formation of oxychloride and that oxychloride formation is inhibited by annealing the CeO_2_ starting material. Lastly, thermal analysis revealed lowering of the melting point of CaCl_2_ after heating with CeO_2_. In all, this work identifies applicable spectroscopic techniques to target studies of heavy elements in molten salt environments and highlights the relationship between chemical speciation and melting behavior, a key thermophysical property.

## Graphical abstract

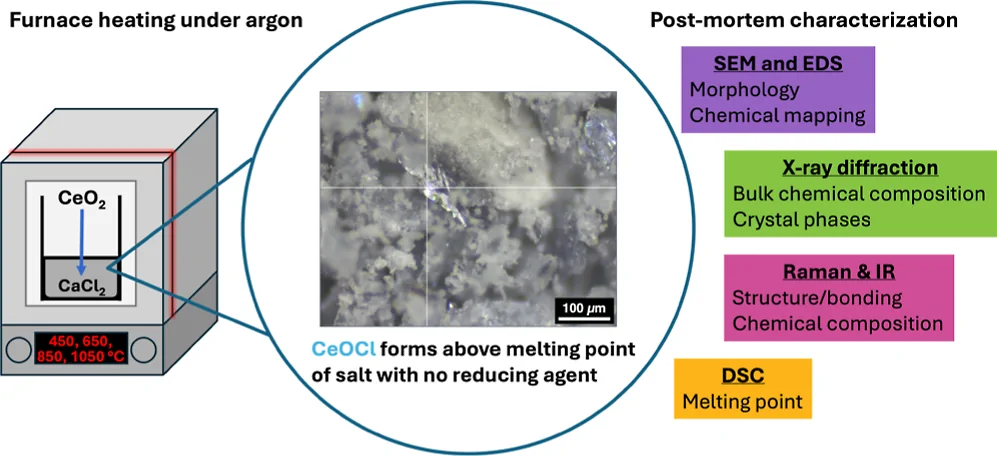

## Introduction

Actinide chemistry has become increasingly integrated with molten salt chemistry. Continued rising interest in the nuclear energy landscape has reinvigorated focus across the industry and national laboratories on molten salt reactors (MSRs) and nuclear fuel cycle separations via pyrochemical processing, which uses molten salt as an electrolyte for reduction.^1–7^ Accordingly, there have been many fundamental studies with actinides in molten salts on thermophysical properties,^8–16^ corrosion interactions,^17–20^ and irradiation effects.^21–24^ Few studies have examined actinide speciation in a molten salt environment; existing work to date is computational, with limited experimental data available to validate predictions.^25,26^ Investigating actinide speciation in molten salts provides information on the chemical species and oxidation state present under molten salt conditions. This information is essential for understanding melting behavior and other thermophysical properties of molten salt systems, with implications for reactor operation, fuel processing, and materials accountability.^27,28^

Experimental studies of actinide speciation in molten salts are hindered by a lack of spectroscopic techniques compatible with high-temperature and radioactive environments. Some groups have implemented UV–visible (UV–vis), infrared (IR), and Raman spectroscopies for high-temperature molten salts.^28–36^ However, these techniques are not broadly available because they typically require custom-made furnaces, and achievable temperatures are constrained by the operating range of the fiber optics used for spectroscopic measurements.^35,37,38^ Additionally, laboratory-scale molten-salt experiments with radioactive uranium (U) and plutonium (Pu), the most common elements used in MSRs and pyrochemical processing studies, are feasible at only a few facilities and are typically limited by material quantities.

To advance speciation analysis capabilities despite these constraints, two complementary strategies can be used. First, post-mortem characterization of samples after heating, while unable to capture transient intermediates and therefore limiting mechanistic insight, can still identify and characterize reaction products. Post-mortem analyses can also be used to screen which spectroscopic methods are sufficiently sensitive to target analytes at relevant concentrations, informing a more targeted campaign to develop in situ, high-temperature spectroscopy. Second, actinide surrogate chemistry enables modeling of actinide behavior without radioactive hazards. Cerium (Ce) is a particularly useful surrogate for U and Pu in the present study due to its similar ionic radius, access to 0, +3, and +4 oxidation states, and similarity in the melting points and crystal structures of the corresponding dioxides.^39–41^

Herein, we report post-mortem characterization of cerium speciation in calcium chloride following heating to operation-relevant temperatures. Samples were analyzed by powder X-ray diffraction (PXRD), scanning electron microscopy (SEM) with energy-dispersive spectroscopy (EDS), and Raman and IR spectroscopies to characterize cerium-containing species and identify methods that are sensitive to the analyte at application-relevant concentrations. Differential scanning calorimetry (DSC) was used to measure the melting point and assess the relationship between cerium speciation and this key thermophysical property.

## Results and Discussion

Cerium dioxide powder (CeO_2_, 200 mg, 1.12 mmol) was ground with calcium chloride powder (CaCl_2_, anhydrous, 500 mg, 4.51 mmol), loaded into an Al_2_O_3_ crucible, and then heated under argon (Ar) in air- and water-free conditions at selected temperatures for 10 h in order to model actinide operation-relevant conditions. The melting point of CaCl_2_ is reported between 772 and 782 °C, and many processes featuring molten CaCl_2_ are heated to ca. 820 °C with potential temperature spikes up to ±100 °C.^11,42^ To capture speciation changes across solid and molten conditions, the samples were heated to 450 °C, 650 °C, 850 °C, and 1050 °C.

After heating, samples were recovered from crucibles. Visual observation reveals distinct changes in color and texture relative to the off-white powder that was initially loaded (Figure 1a). Although 450 and 650 °C are below the melting point of CaCl_2_, both samples exhibited color changes and formed a solidified product rather than free-flowing powders (Figure 1b,c). Both samples were matte solids that adhered strongly to the Al_2_O_3_ crucibles and required mechanical breakup for removal, which resulted in the jagged edges visible in the photographs and required grinding in a mortar and pestle for analysis. The sample heated to 850 °C formed a dark, shiny solid (Figure 1d). In contrast, the sample heated to 1050 °C formed a lighter, shiny product that was heterogeneous, with distinct gray and white regions (Figure 1e). Overall, observed changes in color and the physical form indicate a temperature-dependent interaction between CeO_2_ and CaCl_2_.

**Figure 1:**
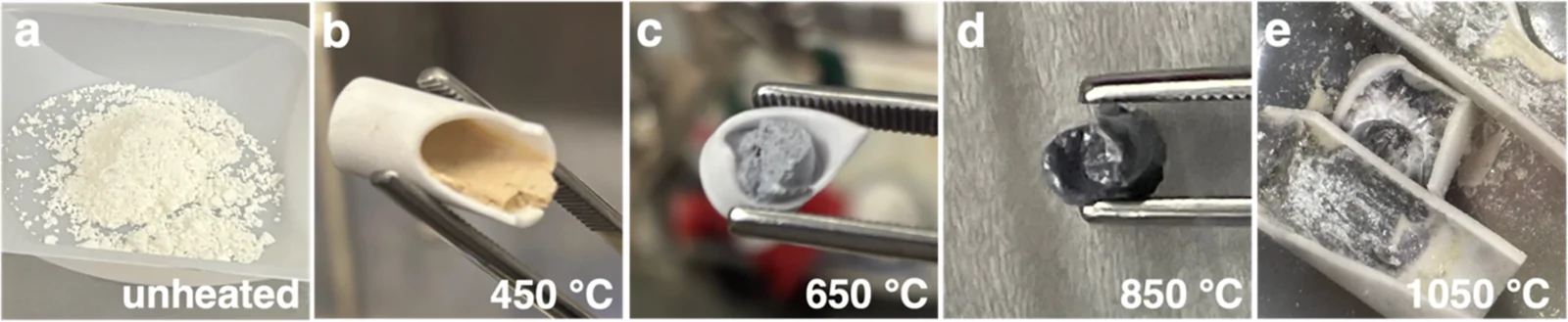
Photographs of CeO_2_–CaCl_2_ samples before heating (a) and after heating to (b) 450 °C, tan product adhered to the crucible wall; (c) 650 °C, light-gray product consolidated into one solidified product at the crucible base; (d) 850 °C, dark-gray, shiny pellet; and (e) 1050 °C, heterogeneous product with shiny-gray and white regions throughout, adhered to the crucible.

### Scanning Electron Microscopy (SEM) with Energy-Dispersive Spectroscopy (EDS)

SEM imaging was performed on products that were ground into fine powders and further reveals morphological changes in the heated samples (Figure 2). In samples heated to 450 and 650 °C, submicron particles (<1 μm) are distributed on the surface of much larger particles. Given the similarity of the samples, only one set of representative images is shown in Figure 2 for both temperatures; Figure 2a depicts the sample under secondary electrons (SE; sensitive to topography), while Figure 2b shows the sample image under backscattering electrons (BSE; sensitive to Z-contrast). Regions with dense clusters of submicron particles tend to charge more under the electron beam, leading to some “smearing” in the images despite carbon coating. The BSE image shows that the submicron particles have higher Z contrast (lighter shading), and these were therefore attributed to Ce-containing phases atop lower-atomic-number Ca-containing material.

**Figure 2:**
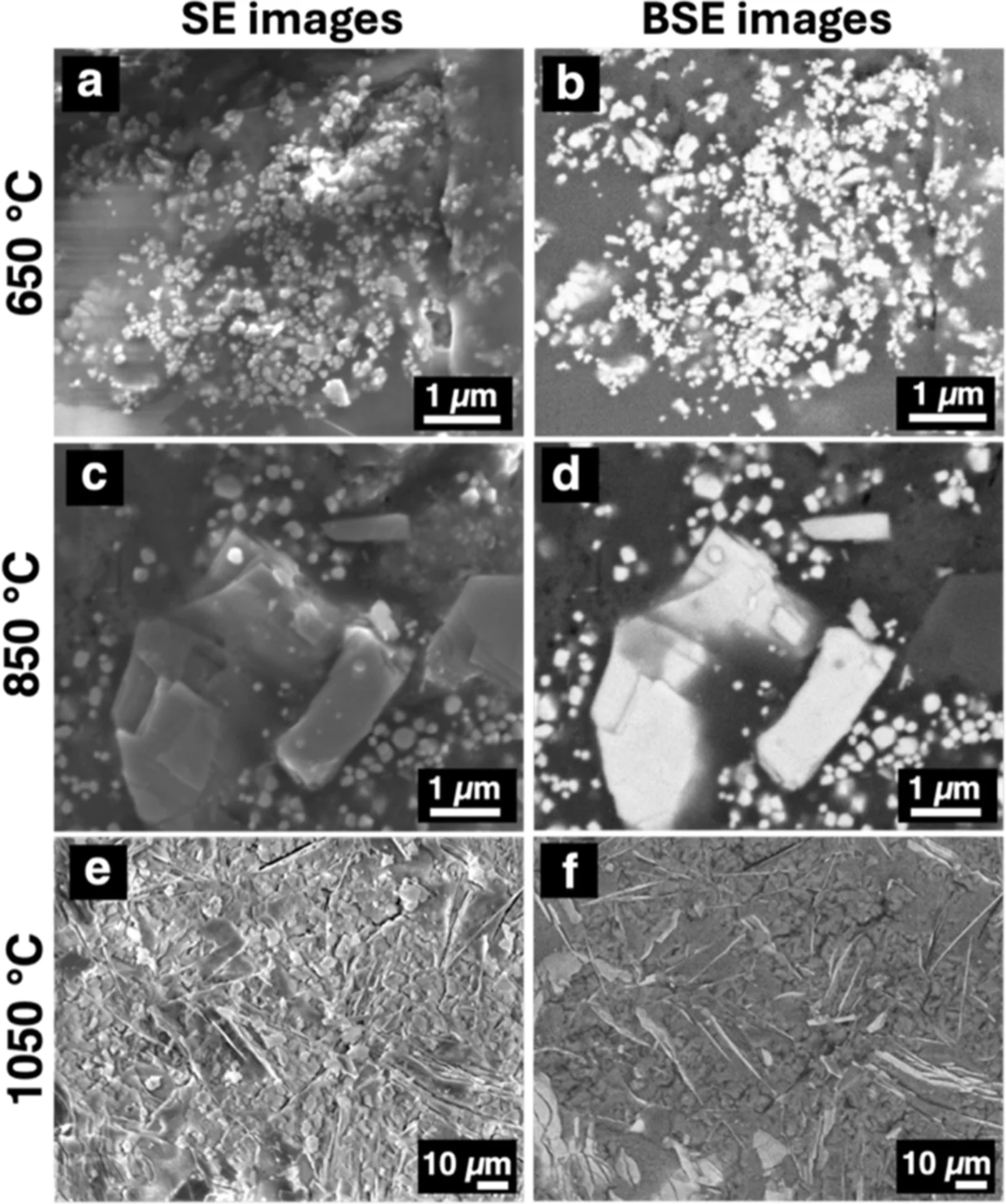
SEM images of CeO_2_–CaCl_2_ samples after heating. Images a, c, and e are secondary electron (SE) images, showing surface topography. Images b, d, and f are backscatter electron (BSE) images, showing Z contrast. Images for the sample heated to 650 °C are shown in (a,b) where submicron Ce-containing particles decorate larger Ca-containing particles; note that samples heated to 450 °C look the same, so they have been omitted for brevity. The sample heated to 850 °C is shown in (c,d), where a mixture of submicron Ce-containing particles and larger Ce-containing sheets coexist. The sample heated to 1050 °C is shown in (e,f), where Ce-containing sheets dominate and appear embedded within larger Ca-containing particles.

Pronounced micron-scale morphological changes are observed only after heating above the CaCl_2_ melting point. In the 850 °C sample (Figure 2c,d), submicron bright particles are still observed, but additional regions have larger Ce-containing sheets approximately 3–5 μm across. The sheets appear to be embedded within, or partially overlapped by, darker Ca-containing regions, indicative of increased interaction between CeO_2_ and molten CaCl_2_. Charging under the electron beam is significant in regions dominated by Ce-containing submicron particles but is reduced in regions where larger sheets are prevalent. Finally, in the sample heated at 1050 °C, submicron Ce-containing particles are not observed; instead, Ce-containing sheets dominate and appear embedded within the larger Ca-containing particles (Figure 2e,f). These narrow sheets extend over longer length scales than those in the 850 °C sample (note the different scale bars), and charging under the electron beam was substantially reduced.

Overall, SEM indicates a temperature-dependent evolution of the Ce-containing morphology from submicron surface-distributed particles (≤650 °C) to embedded sheets at and above the salt melting point (≥850 °C), consistent with enhanced CeO_2_–CaCl_2_ interaction under molten-salt conditions. Given the insolubility of CeO_2_ in CaCl_2_, it is inferred that the observed interactions are a result of chemical changes.^41,43,44^

Initial chemical speciation was assessed using EDS (Figure 3). Mapping was conducted on a sample heated above the melting point of CaCl_2_ since larger Ce-rich agglomerations in these samples improved resolution. The maps show clear separation between Ce-rich (Figure 3b) and Ca-rich (Figure 3c) regions. Chlorine-containing regions (Figure 3d) overlap with Ca-containing regions, consistent with the bulk CaCl_2_ phase from the salt, and are not present within the Ce-rich regions. Oxygen could not be reliably mapped because of its low atomic number and limited spatial resolution in EDS (Figure S1).

**Figure 3:**
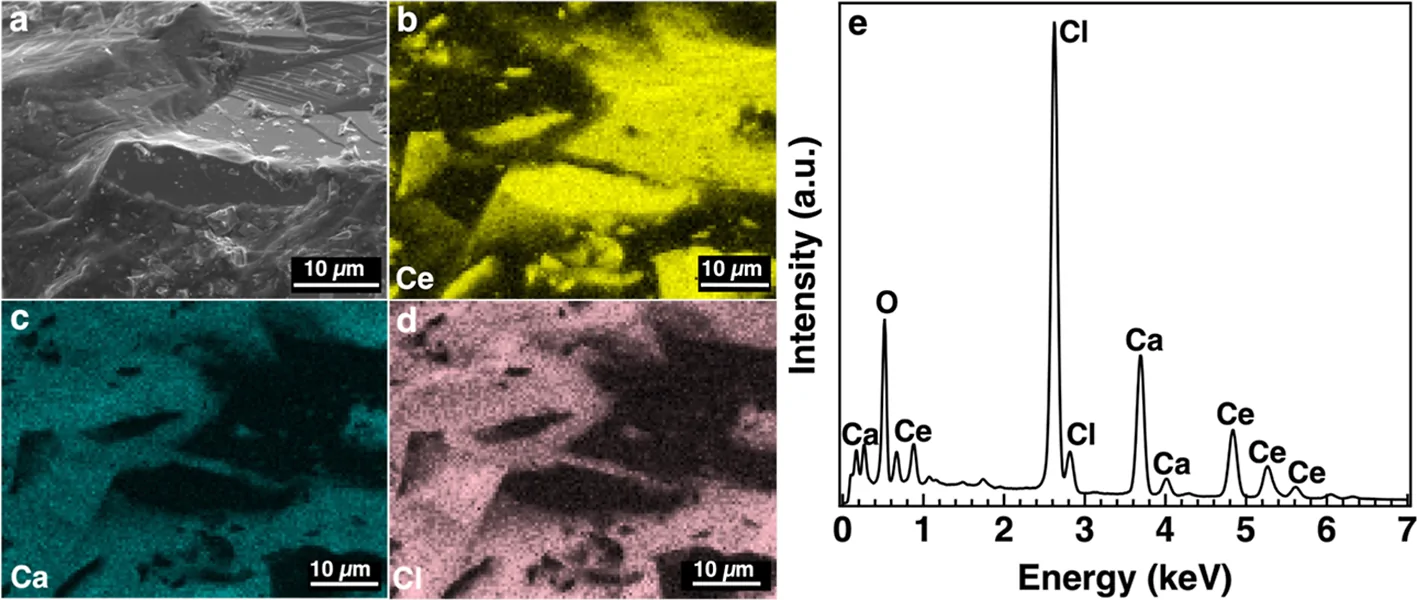
SEM EDS of a representative CeO_2_–CaCl_2_ sample heated to 1050 °C. (a) SE image of the area used for EDS mapping; elemental maps of (b) Ce; (c) Ca; and (d) Cl. These maps show an overlap of Ca and Cl and spatial separation from Ce. Image (e) is a representative EDS spectrum with peaks labeled with corresponding elements; Ca, Ce, Cl, and O. Smaller peaks between 1 and 2 keV and around 6 keV are associated with trace impurities from the experimental crucible and CeO_2_ source material.

The collected EDS spectrum (Figure 3e) shows peaks assigned to Ca (0.34, 3.69, and 4.01 keV), O (0.53 keV), Cl (2.62, and 2.82 keV), and Ce (0.66, 0.88, 4.84, 5.26, and 5.61 keV). Small peaks between 1 and 2 keV and around 6 keV are associated with Al (from the experimental crucible) and trace lanthanides from CeO_2_ manufacturing. Overall, while it is possible to assess which elements are present and the morphology of the regions containing each element, EDS is not sufficient to resolve the bonding interactions or chemical speciation.

### Powder X-ray Diffraction (PXRD)

While not sensitive to trace impurities or noncrystalline phases, PXRD provides information on crystalline phases present in each sample after furnace heating. In samples heated to temperatures below the CaCl_2_ melting point, diffraction patterns of ground products match calculated patterns for CeO_2_ ^45^ and CaCl_2_ ^46^ (Figure 4) and were consistent with experimentally measured starting materials. Additional peaks in the sample index to Al_2_O_3_, which is attributed to mechanical breakage of the crucible to release the sample. No other phases are identified, indicating that, although it underwent a physical change, the bulk of the sample did not chemically react when heated up to 650 °C.

**Figure 4:**
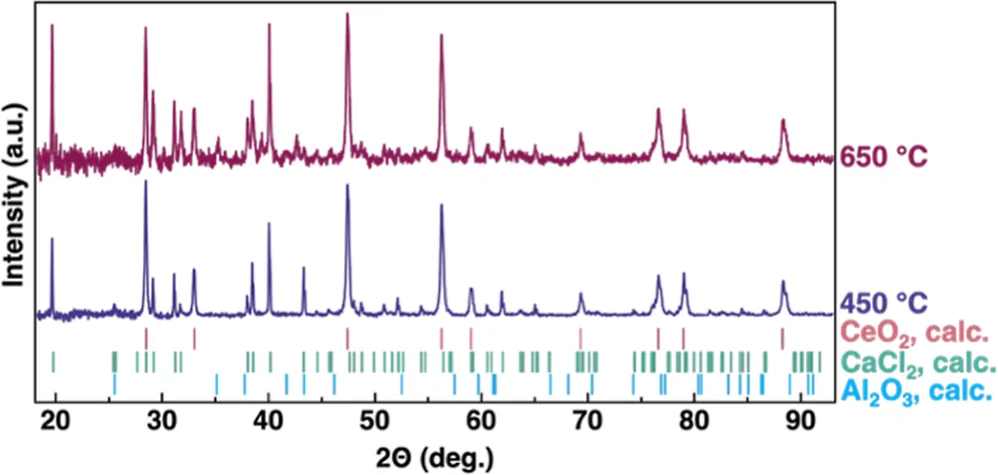
PXRD of CeO_2_–CaCl_2_ samples heated below the melting point of CaCl_2_. The 450 °C sample is shown in dark blue (bottom trace), and the 650 °C sample is shown in maroon (top trace). Experimental patterns are compared with reflections calculated from CIFs of CeO_2_ ^45^ (pink, top row of markers), CaCl_2_ ^46^ (green, middle row of markers), and Al_2_O_3_ ^47^ (light blue, bottom row of markers). All major peaks in the experimental samples align with those of the starting materials and the experimental crucible, without additional unidentified peaks.

All major reflections associated with CaCl_2_ and CeO_2_ (starting materials) and Al_2_O_3_ (crucible) are retained in the samples heated to 850 and 1050 °C, while additional peaks not present in the 450 and 650 °C samples are also observed (Figure 5). When comparing these patterns to other potential species, CeOCl^48^ was identified as the major contributor. In the 850 °C pattern (Figure 5a), peaks indexed to CeOCl are marked with a pink asterisk. There is some overlap between the indicated CeOCl peaks and Al_2_O_3_ diffraction peaks. Diffraction peaks in the 1050 °C pattern (Figure 5b) mimic the assignments in the 850 °C pattern, although with different intensities, indicating more contribution from CeOCl and/or Al_2_O_3_ in the 1050 °C pattern. Different ratios in the marked peaks are attributable to competing contributions between these two species. Of all collected patterns, only the 1050 °C pattern diffracted at angles below 20°; in Figure S2, a strong diffraction peak at 12.9° is visible, which aligns with the [001] orientation of CeOCl. Given the presence of this peak in the 1050 °C sample, but not in the 850 °C sample, and that other CeOCl-associated peaks increased in intensity, it can be concluded that the CeO_2_–CaCl_2_ mixture reacted further at higher temperature to produce more crystalline CeOCl.

**Figure 5:**
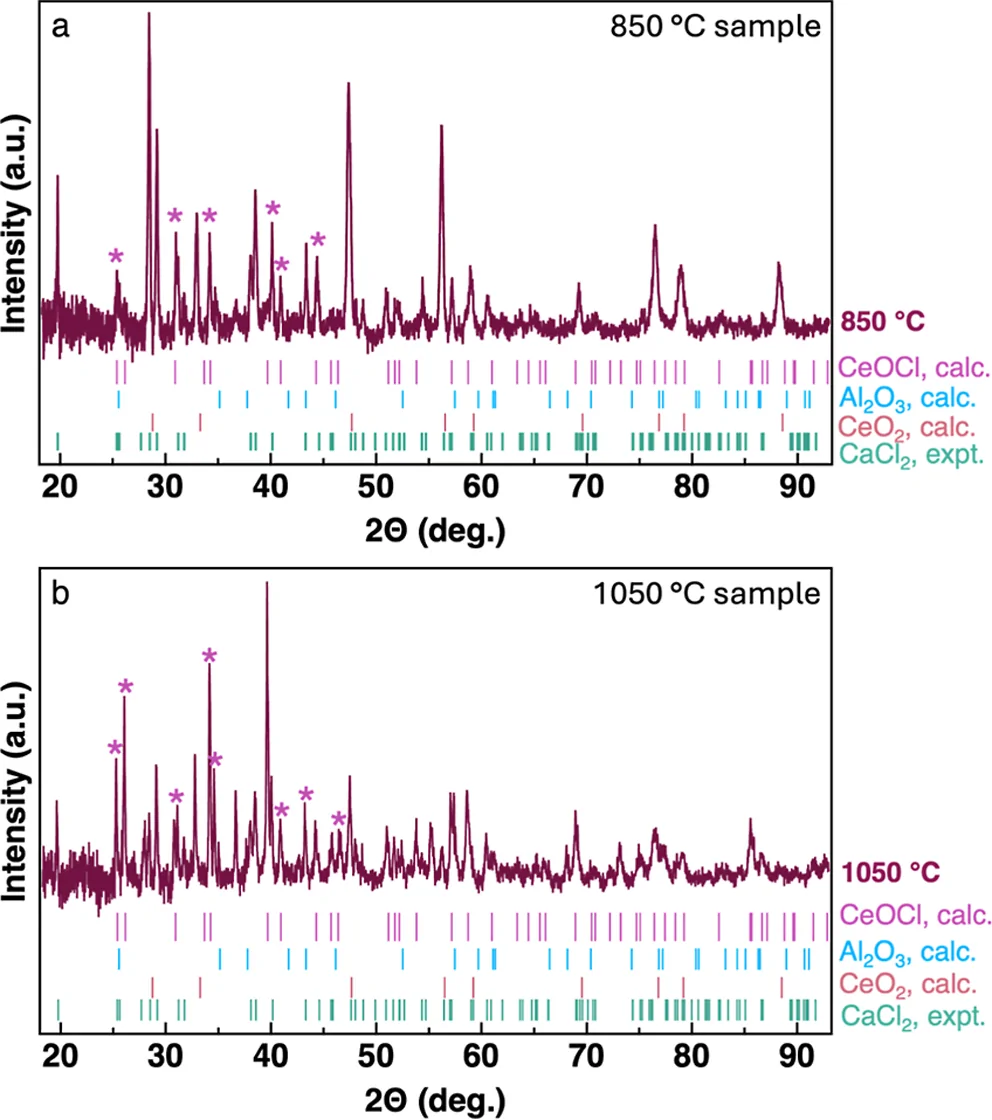
Powder patterns for samples heated above the melting point of CaCl_2_. The pattern for a sample heated to 850 °C (a, top) compares well to experimental CaCl_2_ (green, bottom row of markers) and calculated CeO_2_ (light red markers)^45^ and Al_2_O_3_ ^47^ (light blue markers). Additional peaks are consistent with the calculated patterns for CeOCl (bright pink, top row of markers);^48^ specific peaks indexed to CeOCl are marked with a pink asterisk. 1050 °C samples (b, bottom) similarly compare well to experimental starting materials, and extraneous peaks (marked with a pink asterisk) match CeOCl.

This interpretation was further assessed by performing Rietveld refinement on experimental patterns using GSAS-II software,^49^ the results of which are presented in Table 1. The sample heated to 850 °C is well-described by a model with the starting materials, CaCl_2_ and CeO_2_. The refinement is further improved by the addition of CeOCl and Al_2_O_3_. Similarly, the sample heated to 1050 °C contains both starting materials as well as CeOCl and Al_2_O_3_. Contributions from Al_2_O_3_ are attributable to mechanical breakage of crucibles for sample recovery when salt mixtures adhered to them, and there is no evidence of chemical interaction between the crucible and CeO_2_ (Figure S3).^47,50^ Overall, these models show a decrease in CeO_2_ and a corresponding increase in crystalline CeOCl contribution with increasing temperature. Additional cerium species, such as reduced oxide Ce_2_O_3_, were modeled but did not match the powder pattern for either sample (Figure S3).^51^ Previous work on cerium in molten chloride salts indicates the stability of the oxychloride over the sesquioxide.^52–54^ No other peaks or species were required to model the patterns for these products, indicating that there are no other bulk crystalline components present.

**Table 1:** Results of Rietveld Refinement for Samples Heated at 850 and 1050 °C; Fit Calculations on Experimental Samples Were Conducted against CIFs of CaCl_2_,^46^ CeO_2_,^45^ CeOCl,^48^ and Al_2_O_3_.^47^

Species	850 °C Wt %^a^	1050 °C Wt %^b^
CaCl_2_	54	38
CeO_2_	21	6
CeOCl	10	23
Al_2_O_3_	15	33

a Χ^2^ = 4.4, w*R* % = 3.62.

b Χ^2^ = 8.13, w*R* % = 4.5.

The reaction to form CeOCl is somewhat unexpected in this system without the identification of a reducing agent in the salt mixtures and no application of an electrochemical potential in these experiments. In CeO_2_, Ce is in the +4 oxidation state, while in CeOCl, it is in the +3 oxidation state. When intentionally targeted, CeOCl is typically made from CeCl_3_ (a Ce^3+^ material) as a starting material, with oxygen introduced by materials such as CeO_2_ or MgO.^55,56^ Alternatively, CeOCl has been made from CeO_2_ in the presence of hydrogen and metal chloride catalysts, where hydrogen creates a reducing atmosphere.^57,58^ In many cases where CaCl_2_ is used as a molten solvent, the presence of CaO from the salt drying process will lead to reduction of metal oxides with the application of electrolytic potential.^42,59,60^ No possible solid chemical reducing agent was added to the reaction nor identified in PXRD of the starting materials. Other crystalline chemicals were not identified in the patterns for any of the heated products, indicating that additional spectroscopic methods are required to identify a potential reducing agent.

### Infrared (IR) Spectroscopy

Recovered samples were ground in a mortar and pestle to form homogeneous, free-flowing powders, which were then analyzed by IR spectroscpoy (Figure 6). IR spectra for all samples show strong absorption bands associated with CaCl_2_ in the range of 1613–1645 cm^–1^ and 3200–3600 cm^–1^.^61^ Weak peaks between 2100 and 2400 cm^–1^ are assigned to CeO_2_.^62,63^ Peaks associated with Al_2_O_3_, which were identified by PXRD, generally overlap with CaCl_2_ absorption peaks and therefore cannot be reliably distinguished by IR spectroscopy.^64,65^

**Figure 6:**
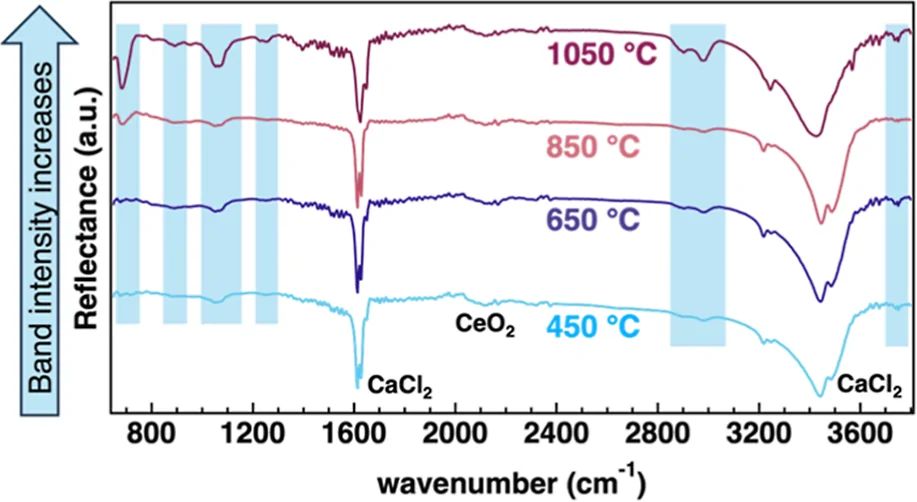
Reflectance IR spectra of CeO_2_–CaCl_2_ samples after heating to 450 °C (light blue), 650 °C (dark blue), 850 °C (pink), and 1050 °C (red). Spectra were normalized to the intensity of the peak at 1613 cm^–1^, which is associated with CaCl_2_. The other peak associated with CaCl_2_ is the broad stretch between 3200 and 3600 cm^–1^.^61^ Very weak peaks around 2100 cm^–1^ are associated with CeO_2_.^62,63,66^ Peaks highlighted in light blue are associated with Ce–O vibrations and are highlighted because they change with heating of the sample, indicating a change in the bonding and therefore speciation of the sample. Given the association with Ce–O stretching, these peaks are attributed to the formation of CeOCl in higher-temperature samples.

Across the series from the lowest to highest temperature, several peaks increase in intensity. Peaks at approximately 682, 887, 1058, 1241, 2896, 2978, and 3735 cm^–1^ are weak or absent in the 450 and 650 °C samples but become more prominent in the 850 °C sample and even more intense in the 1050 °C sample. These bands are generally associated with Ce–O vibrational modes.^62,63,66^ Increasing band intensity suggests changes in cerium bonding, leading to changes in cerium speciation with increasing temperatures.^62^

A sample of CeOCl on its own was measured (Figure S4) and shows broad peaks centered around 3240 cm^–1^ and 1036 cm^–1^ and narrower peaks at 716, 1610, 2296, and 2091 cm^–1^. The broad peak at 3240 cm^–1^ overlaps with the 3200–3600 cm^–1^ stretch for CaCl_2_; in higher-temperature samples of the CeO_2_–CaCl_2_ mixture, this CaCl_2_ feature is broadened and loses a double peak feature. This indicates that CeOCl’s contribution to the mixture can additionally be tracked by the broadening of this peak. Similarly, the change in the peak shape for the 1613 cm^–1^ CaCl_2_ peak in the higher-temperature samples can be attributed to CeOCl. Finally, the broad feature around 1036 cm^–1^ likely contributes to the increased intensity of the lower-wavenumber peaks in the high-temperature CeO_2_–CaCl_2_ sample.

Importantly, there are no unidentified bands, indicating that a possible reductant for CeO_2_ to CeOCl is not identifiable with post-mortem IR characterization.

### Raman Spectroscopy

Ground samples were additionally studied with Raman spectroscopy, which is sensitive to different vibrational modes than IR (Figure 7). Raman microscopy specifically allows for spatial resolution and sampling of different regions, rather than only bulk measurements. Microscopic examination (Figure 7a) reveals three distinct regions in the 1050 °C sample. The “salt region” appears predominantly white without other visible features and is assumed to be primarily CaCl_2_. Raman analysis of this region reveals weak bands between 50 and 200 cm^–1^, consistent with literature spectra for CaCl_2_.^67,68^ Additional low-intensity, broad features are observed and can be assigned to CeOCl (between 100 and 200 cm^–1^ and 327 cm^–1^) and CeO_2_ (458 cm^–1^).^69–71^ Since CaCl_2_ and CeOCl bands overlap in the 100–200 cm^–1^ range, this region is not diagnostic for distinguishing between the two phases. However, the band at 327 cm^–1^ is unique to CeOCl and can be used as a marker for this species.^69^ A band at 409 cm^–1^ that would be associated with Ce_2_O_3_ was not observed, indicating that this species is not present in this sample.^72^ Raman spectra collected in the “colored region” (a mixture of white and gray particles) contain the same stretches, with higher relative intensity from the CeO_2_ peak, indicating that the coloration originates from Ce-containing particles. Spectra from the “shiny region” (a solid gray, shiny particle) are dominated by a very strong CeO_2_ band, with additional contribution from CeOCl.

**Figure 7:**
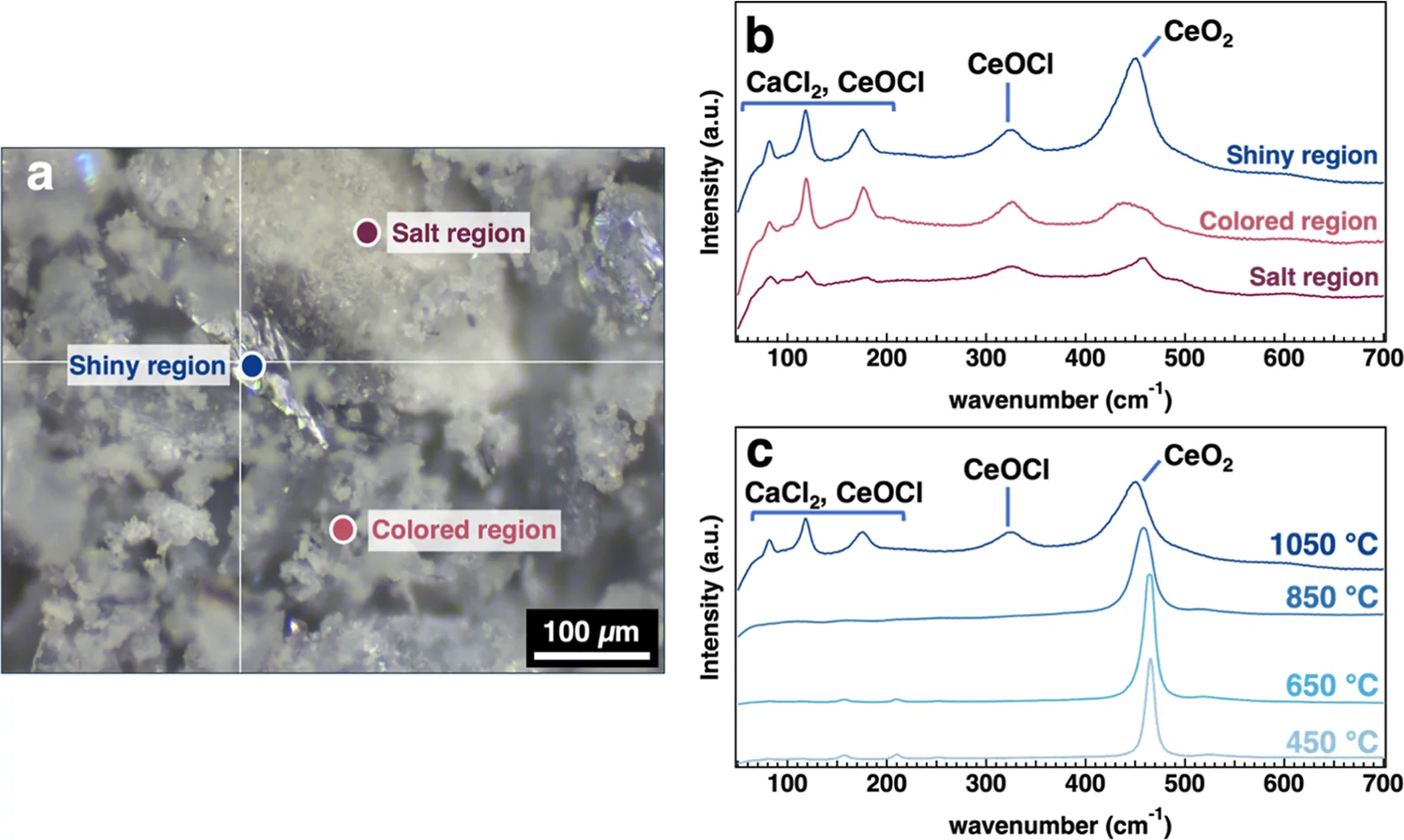
Spatially resolved Raman spectra of heated CeO_2_–CaCl_2_ samples. (a) Example microscopy image used to select differing regions. The “salt region” (dark red) is from a predominantly white region of the sample; the “colored region” (light red) is from a gray-white mixed region of the sample; and the “shiny region” (blue) is from a gray, shiny particle. (b) Raman spectra collected from the distinct regions of the 1050 °C sample as marked in (a). (c) Raman spectra collected after heating the samples to 450 °C, 650 °C, 850 °C, and 1050 °C. Spectra in (c) were collected on “shiny” regions of the sample or, for lower-temperature samples, from regions with comparable CeO_2_ band intensity. Stretches in (b,c) are labeled with CaCl_2_ from ca. 50 to 200 cm^–1^;^67,68^ CeOCl from 100 to 200 cm^–1^ and 300 to 360 cm^–1^;^69^ and CeO_2_ from 400 to 500 cm^–1^.^70,71^ Spectra were collected out to 1600 cm^–1^, but no additional bands were observed after 700 cm^–1^, and these data were omitted.

Comparison of Raman spectra for the four heated samples reveals changes in the band shape and intensity with increasing temperature. As the sample is heated above the melting point of CaCl_2_, the band at 458 cm^–1^, attributed to CeO_2_, loses intensity and broadens. This is consistent with the loss of CeO_2_ content observed in the Rietveld refinement of PXRD for these samples. It has also been reported that, when partially reduced, CeO_2_ has a peak at 459 cm^–1^, while fully oxidized CeO_2_ produces a Raman peak at the slightly higher wavenumber of 465 cm^–1^.^73^ Raman spectra shown in Figure 7b show a slight shift downward in peak position as the sample is heated to higher temperatures, indicative of contributions from Ce^3+^ in cerium speciation. While the Raman spectrum for the 1050 °C sample has a band assignable to CeOCl at 327 cm^–1^, Raman spectra for the 450 °C, 650 °C, and 850 °C samples do not contain this band, indicating the lack of CeOCl in these samples. In the case of the 850 °C sample, CeOCl was identified by PXRD, indicating that the lack of a band in Raman spectra is a result of the lower CeOCl concentration in this sample or of lower crystallinity of CeOCl when heated to 850 °C, as indicated by the less intense peaks in the powder pattern for this sample. Lastly, low-frequency bands in the 1050 °C sample appear stronger than in other samples; this is likely a result of increased CeOCl contribution to this region. There were no unidentifiable peaks or peaks that disappear with heating to higher temperatures, indicating that there are no other characterizable species present in the sample.

All characterization methods indicated that CaCl_2_, CeO_2_, CeOCl, and Al_2_O_3_ were the only species present in CeO_2_–CaCl_2_ samples after heating. Although the presence of CeOCl implies a reduction of Ce in CeO_2_ from Ce^4+^ to Ce^3+^, no chemical reducing agent was detected in appreciable quantities. Previous studies of CeO_2_ behavior have indicated that high temperatures and low-oxygen environments promote oxygen loss to the gas phase, producing point defects when the sample is at high temperature that are charge-balanced by Ce^3+^ electronic defects, often producing Ce_2_O_3_.^40,73–76^ Additionally, reports using CeO_2_ as a starting material for CeOCl have shown that a reducing environment creates oxygen vacancies on the surface of CeO_2_, which are then filled by chlorine ions.^57,58^ In this study, CeOCl formation was first observed after heating to 850 °C under flowing UHP Ar gas, suggesting that the high-temperature, low-oxygen environment promotes the formation of oxygen vacancies in CeO_2_, leading to the presence of Ce^3+^ in the molten salt. These vacancies are then filled by Cl^–^ from the CaCl_2_ salt in the system, leading to the formation of a stable Ce^3+^ species, CeOCl. By contrast, even with a low-oxygen environment, experiments run at 650 and 450 °C did not form CeOCl, implying that elevated temperature, not low oxygen partial pressure alone, is necessary for oxygen vacancy formation and subsequent CeOCl generation.

The role of defects in CeOCl formation was further probed by annealing the CeO_2_ starting material as surface properties of CeO_2_ have been shown to depend on annealing temperature.^77,78^ CeO_2_ was annealed at 920 °C for 3 h under Ar, followed by the same CeO_2_–CaCl_2_ experiment at 1050 °C for 10 h. PXRD of this product matches powder patterns of the starting CaCl_2_ and CeO_2_, with particularly strong diffraction from CeO_2_ (Figure 8). There are no additional contributions from CeOCl. These results indicate that annealing of CeO_2_ prior to molten salt treatment suppresses CeOCl formation, consistent with a reduction in defect density and oxygen vacancies, and thus alters the conditions that promote oxychloride formation. In all, this result highlights the importance of material defects in influencing speciation of the final product; reducing defects in starting material can suppress oxychloride formation in this system.

**Figure 8:**
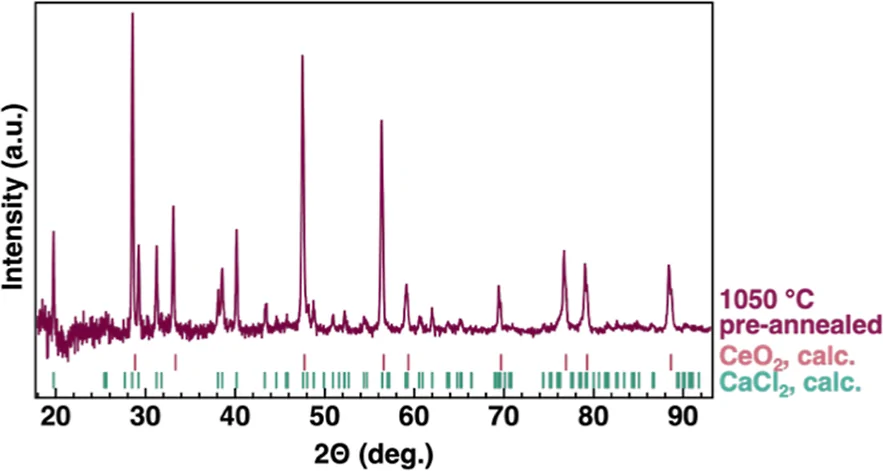
PXRD of CeO_2_–CaCl_2_ heated to 1050 °C after CeO_2_ was annealed at 920 °C for 2 h. The sample is shown in red and compared to reflections calculated from CIFs of CeO_2_ ^45^ and CaCl_2_.^46^ All major peaks in the experimental sample align with those of the starting materials, without additional unidentified peaks.

### Differential Scanning Calorimetry (DSC)

Changes in speciation are expected to result in corresponding changes in physical properties of the mixture. For molten salt applications using CaCl_2_, the melting point is a particularly relevant property for operational parameters. Differential scanning calorimetry (DSC) was conducted on CaCl_2_ alone and on a CeO_2_–CaCl_2_ sample previously heated to 1050 °C (Figure 9a). Both samples were heated under Ar to 823 °C, allowed to cool, and then heated in a second cycle to 823 °C. The DSC profile for CaCl_2_ shows an exothermic peak with a maximum of 782 °C, consistent with the reported melting point of 772–782 °C.^11,42^ In contrast, the heated CeO_2_–CaCl_2_ mixture exhibits an exothermic peak with a maximum temperature of 753 °C. This is distinctly lower than that of pure CaCl_2_, consistent with melting point depression in a multicomponent system. An additional CeO_2_–CaCl_2_ sample that was not previously heated was also studied, with DSC following the same heating profile (Figure 9b). The first heating cycle shows one exothermic peak with a maximum at 778 °C, while the second cycle shows two distinct peaks with maxima at 751 and 775 °C. The peak at 751 °C aligns with the lower temperature peak observed in the sample heated to 1050 °C, while the peak at 775 °C falls within the range of melting for CaCl_2_. This indicates that the change in the melting point of the system is not attributable only to mixing CeO_2_ with CaCl_2_ but is associated with CeOCl formation.

**Figure 9:**
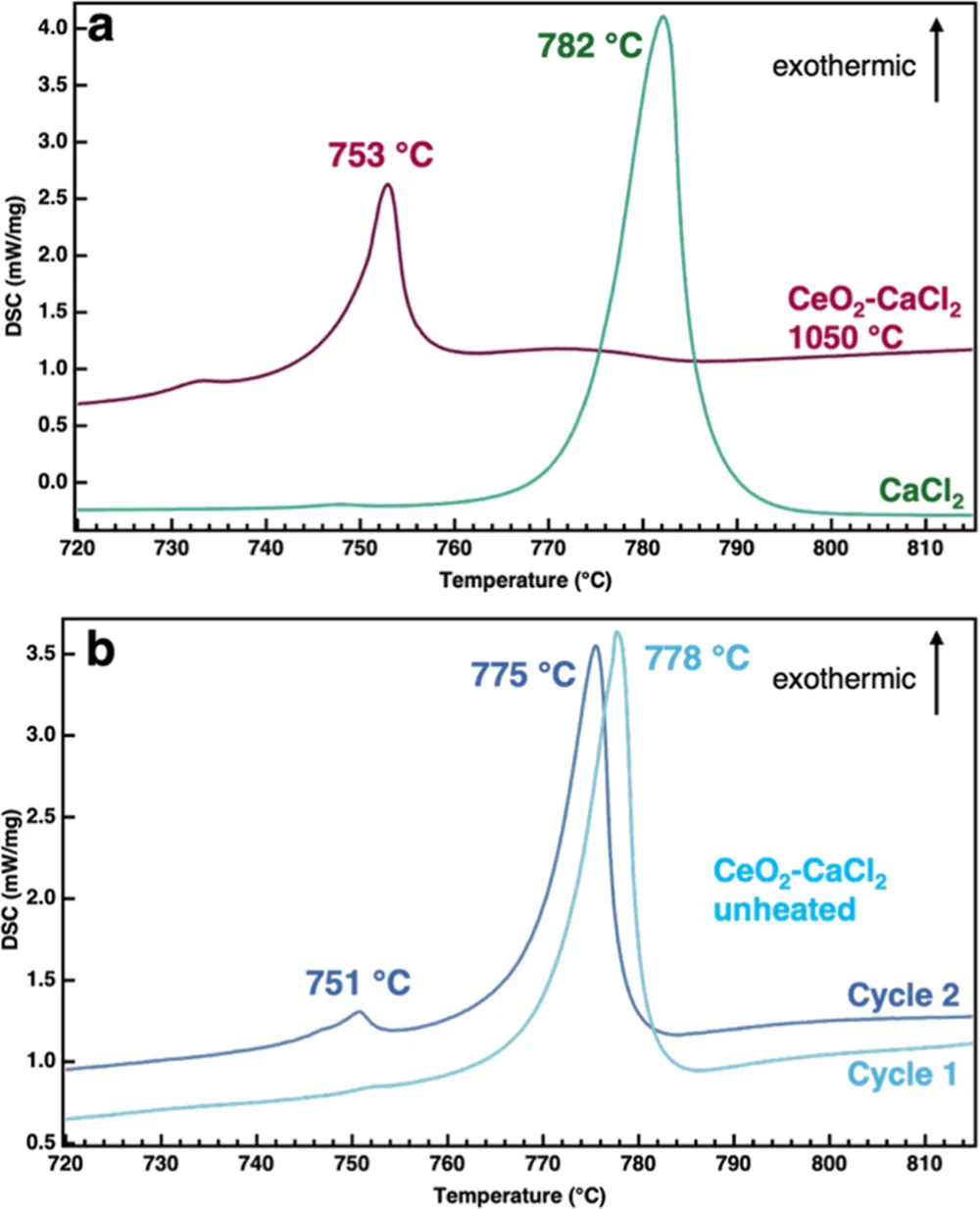
Differential scanning calorimetry of (a) pure CaCl_2_ (green); compared to the CeO_2_–CaCl_2_ mixture that was previously heated in a furnace to 1050 °C for 10 h (red). For DSC, both samples were heated to 823 °C, cooled, and heated again. CaCl_2_ has an exothermic peak, representing melting of the sample, with a peak at 782 °C, matching the reported melting point for CaCl_2_. The CeO_2_–CaCl_2_ mixture has an exothermic peak at 753 °C, which is significantly lower than CaCl_2_’s melting point. (b) Two heating cycles for a previously unheated mixture of CeO_2_–CaCl_2_. Cycle 1 (light blue) has a peak at 778 °C, within the range of CaCl_2_’s melting point. Cycle 2 (dark blue) has a slightly lower peak at 775 °C, as well as a second peak at 751 °C, near the observed peak for the sample heated to 1050 °C.

## Conclusion

This work reports changes in cerium speciation in molten calcium chloride following heating above and below the melting point of the salt, along with corresponding changes in melting behavior. The importance of starting material crystallinity and defects in controlling the reactivity of the metal oxide is also highlighted. In general, heating to temperatures below the melting point of CaCl_2_ (ca. 772 °C) does not affect cerium speciation, as shown by PXRD, IR, and Raman spectroscopy. When heating to a typical operating temperature, 850 °C, some formation of CeOCl is observed by PXRD and IR spectroscopy; Raman spectroscopy is not sensitive to this species at low concentrations. Further formation of CeOCl is observed after heating to 1050 °C, supported by Rietveld refinement of PXRD, as well as IR and Raman spectroscopy and increased interaction with the salt matrix as observed by SEM. Possible reducing agents for the change in the Ce-oxidation state from +4 (CeO_2_) to +3 (CeOCl) are not observed by spectroscopic methods. Instead, the high-temperature, low-oxygen environment is proposed to promote the formation of oxygen vacancies and electronic defects that then contribute to CeOCl formation. An annealed, and therefore more crystalline, CeO_2_ sample did not produce CeOCl, supporting the importance of defect formation at high temperature in generating metal oxychloride species. Lastly, measurement of the melting points of previously heated and unheated CeO_2_–CaCl_2_ mixtures indicates that changes in speciation are associated with changes in melting behavior, a key thermophysical property, with a ca. 30 °C decrease in melting point associated with CeOCl formation. This study identifies materials characterization techniques that are sensitive and applicable to actinide-molten salt chemistry and that can be used to develop in situ characterization for molten salts at temperatures above 800 °C. These results demonstrate the importance of speciation and defect structure in governing reactivity and thermophysical behavior in molten salt systems.

## Experimental Section

### Methods and Materials

General considerations: Unless otherwise noted, all reactions were performed under an atmosphere of argon, either in an inert atmosphere glovebox or in furnaces plumbed with argon lines. Glassware was heated in an oven ca. 100 °C for at least 12 h prior to use. Alumina (Al_2_O_3_) crucibles were dried in a box furnace at 500 °C for 8 h prior to use.

There are no uncommon hazards noted.

### Materials

Calcium chloride used at Lawrence Livermore National Laboratory (Alfa Aesar, anhydrous, ampule packed under Ar) was opened and stored in an inert atmosphere glovebox. At Los Alamos National Laboratory, calcium chloride (Sigma-Aldrich) was dried under a vacuum in a glovebox antechamber equipped with a heater prior to use. Cerium dioxide (Fisher Scientific) was used as received. Cerium oxychloride was synthesized according to literature procedures.^56^

### Furnace Experiments

In an Ar-filled glovebox, approximately 200 mg (1.12 mmol) of CeO_2_ powder was ground in a mortar and pestle with approximately 500 mg of CaCl_2_ (4.51 mmol) until a fine off-white homogeneous powder was formed. This salt mixture was loaded into an alumina crucible and placed into a controlled atmosphere muffle furnace. The furnace was pumped/purged under Ar and vacuum thrice before heating under a flowing Ar atmosphere. The temperature was raised to the desired temperature (450 °C, 650 °C, 850 °C, or 1050 °C) over the course of 1 h, then held at temperature for 10 h, and finally cooled to room temperature. The sample was then quickly moved back into the glovebox for sample preparation and storage. Powder samples were stored in scintillation vials. Solid samples that were adhered to crucibles were scraped or smashed and ground into powders before storing in scintillation vials.

To anneal CeO_2_, 1.5 g of CeO_2_ was loaded into an Al_2_O_3_ crucible and then heated in an Ar-filled furnace at 920 °C for 2 h. The sample was recovered and returned to an inert atmosphere glovebox for storage.

### Powder X-ray Diffraction (PXRD)

Samples were collected in an inert atmosphere on a zero-background Si plate PXRD holder. The samples were then covered with Kapton tape and sealed. A D2 Phaser X-ray powder diffractometer (Bruker) with a Cu anode was used to collect PXRD patterns at 30 kV and 10 mA power. A typical PXRD pattern was collected in the 10–100° 2θ range with a step size of 0.026° and 1 s per step. The data were analyzed by Rietveld refinement using the general structure analysis system (GSAS).^49^

### Infrared (IR) Spectroscopy

Solid samples were placed on a Cary 630 FTIR Spectrometer with an ATR (diamond) sample module outside of an inert atmosphere. Powders were placed on the sample stage, and spectra were collected in triplicate from 650 to 4000 cm^–1^.

### Raman Spectroscopy

Solid samples were placed in a custom holder with an alumina stage and enclosed to be airtight with a quartz window atop the sample to be imaged through. An Edinburgh Instruments RM-5 microscope was used with a 532 nm laser and a 10× objective to collect spectra in the dark-field mode. The Raman shift was calibrated with an internal silicon standard prior to each collection.

### Scanning Electron Microscopy (SEM) and Energy-Dispersive Spectroscopy (EDS)

SEM samples were prepared by pressing ground solid samples onto carbon tape on an SEM stub and then coating with 10 nm of carbon to avoid or reduce charging on the samples during imaging. Samples were transported in air to both the coating instrument and the SEM setup before being quickly loaded and placed under vacuum. Data were collected on an Apreo S (Thermo Fisher Scientific, Pleasanton, CA) SEM setup equipped with an EDAX Octane Elite Plus silicon drift detector.

### Differential Scanning Calorimetry (DSC)

Samples of ∼5–20 mg were loaded into alumina crucibles in a glovebox and then transferred in air to the instrument. Prior to sample analysis, the instrument was calibrated with a series of metal calibrants. DSC was performed on a NETZSCH 404 F1 Pegasus. The sample chamber was purged and flushed thrice to ensure an inert atmosphere prior to sample heating. Samples were heated to 823 °C at a constant heating rate of 10 °C/min, held at this temperature for 5 min, and then cooled to 500 °C before repeating this cycle.

## Supplementary Material



## Notes

Additional EDS, PXRD, and IR data (PDF)
