# Supplementary material for: Post-Mortem Characterization of Cerium Speciation in Molten Calcium Chloride
Source: Inorg Chem. 2026 Sep 9;65(37):21523–32. doi: 10.1021/acs.inorgchem.6c02415 (PMC13599382; doi:10.1021/acs.inorgchem.6c02415)
Supplement: Supplementary file 1 [file ic6c02415_si_001.pdf]

Supplementary Information

for:

Post-mortem characterization of cerium speciation in molten calcium chloride

Sheridon N. Kelly,[a,b] Alfred Amon,[a] Maryline G. Ferrier,[a] Karla A. Erickson,[b] L. Eddy Banks,[a] Marisa J. Monreal,[b*] Bradley C. Childs[a*]

[a] *Physical and Life Sciences Directorate, Lawrence Livermore National Laboratory, Livermore, CA 94550 USA*

[b] *Chemistry Division, Los Alamos National Laboratory, Los Alamos, NM 87545 USA*

**Email: childs4@llnl.gov; mmonreal@lanl.gov*

Table of Contents

A. Energy Dispersive X-Ray Spectroscopy (EDS)…………………………………………...S2

B. Powder X-Ray Diffraction (PXRD)…..…………………………………………..……..S2

C. Infrared (IR) Spectroscopy…...……………………..…………………………...……..S4

D. References…………………………………………………………………………….S4

A. Energy Dispersive X-Ray Spectroscopy (EDS)

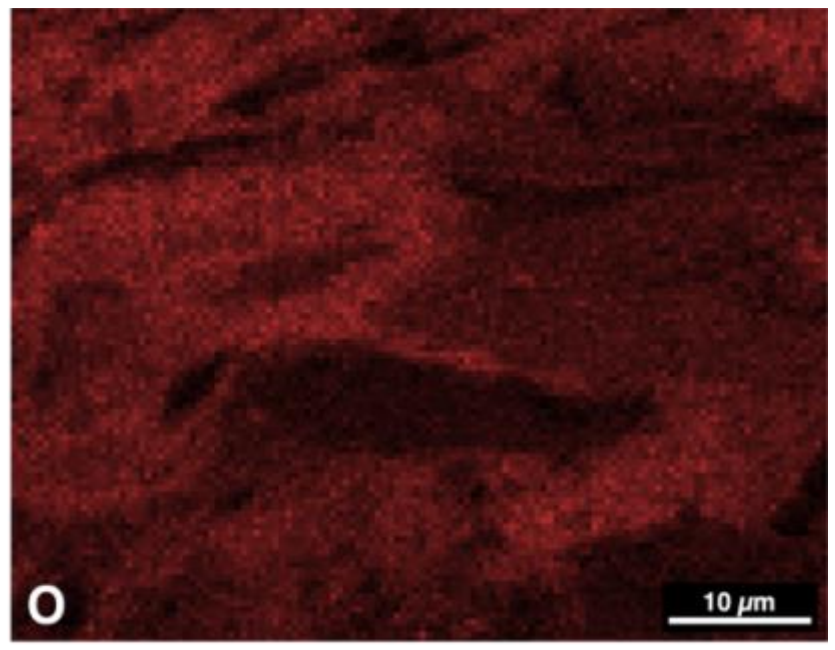

Figure S1. SEM EDS elemental map of oxygen in a representative CeO_2-$CaCl_2$ sample. While oxygen is too light to quantitatively resolve with EDS, it is visible in mapping and can be seen throughout the sample.

B. Powder X-Ray Diffraction (PXRD)

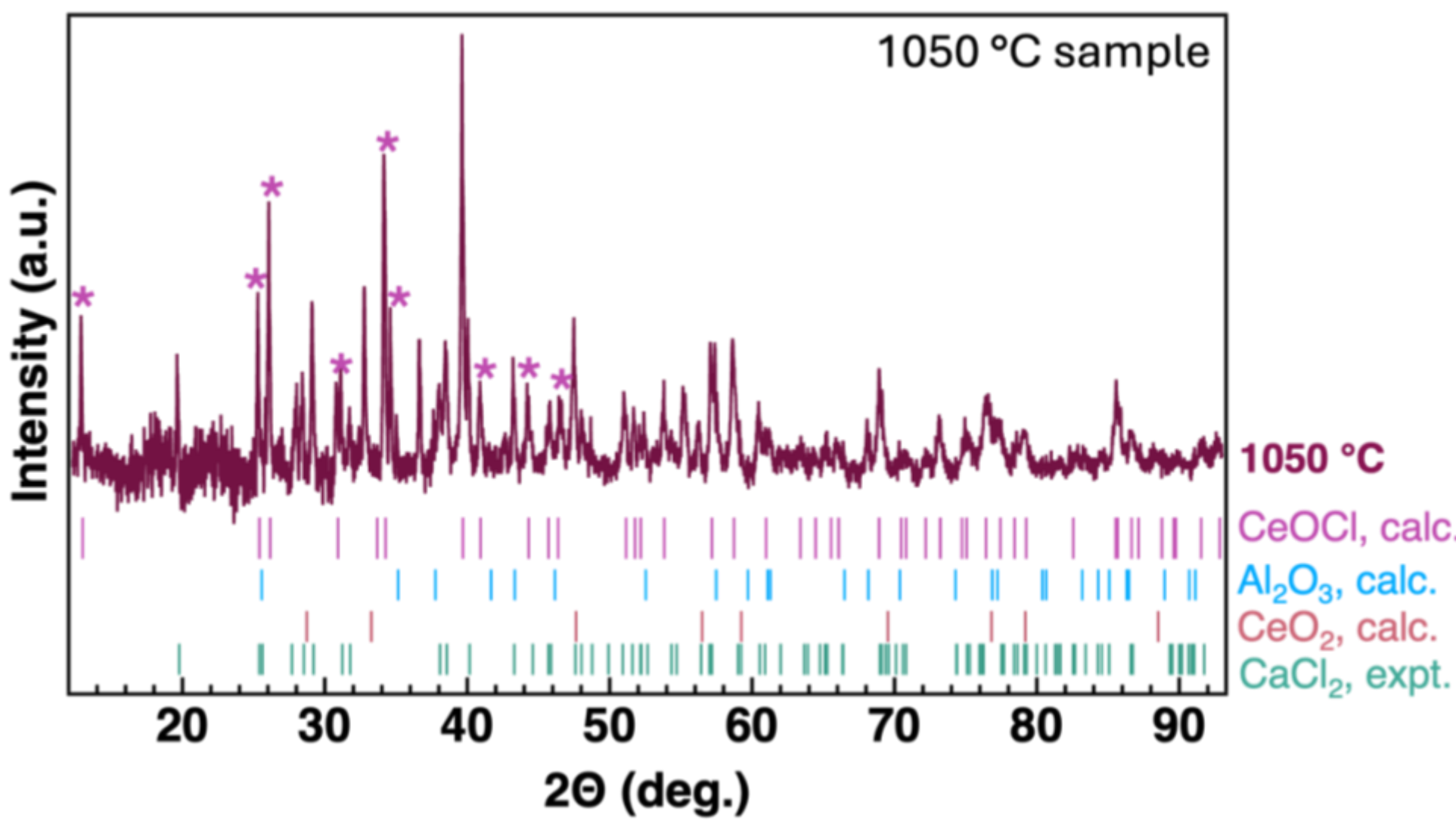

Figure S2. PXRD for sample heated to 1050 °C, with diffraction measured down to 10°. This is the only sample with a diffraction peak at ~12.9°, a peak associated with the [001] orientation for CeOCl.[1]

Table S1. Rietveld refinement for CeO_2-$CaCl_2$ samples heated to 450 °C and 650 °C.

Species	450 °C Wt%[a]	650 °C Wt%[b]
$CaCl_2$[2]	48.5	53
CeO_2[3]	45.7	37
Al_2O_3[4]	5.8	10

[a]X^2 = 11.78, wR% = 5.759; [b]X^2 = 7.869, wR% = 4.879

Table S2. Rietveld refinement results for CeO_2-$CaCl_2$ samples heated to 850 °C and 1050 °C, showing models with starting materials only, addition of Al_2O_3, and finally addition of CeOCl to demonstrate improvement of statistics as additional phases are modeled.

Sample + fit	X^2	wR%	CeO_2[3] wt%	$CaCl_2$[2] wt%	Al_2O_3[4] wt%	CeOCl[1] wt%
850 °C, $CaCl_2$+CeO_2 only	5.7	4.10	26	74	N/A	N/A
850 °C, +Al_2O_3	5.6	4.07	22	64	14	N/A
**850 °C, +CeOCl**	**4.4**	**3.62**	**21**	**54**	**15**	**10**
1050 °C, $CaCl_2$+CeO_2 only	12.9	5.64	13	87	N/A	N/A
1050 °C, +Al_2O_3	12.3	5.52	8	48	44	N/A
**1050 °C, +CeOCl**	**8.13**	**4.5**	**6**	**38**	**33**	**23**

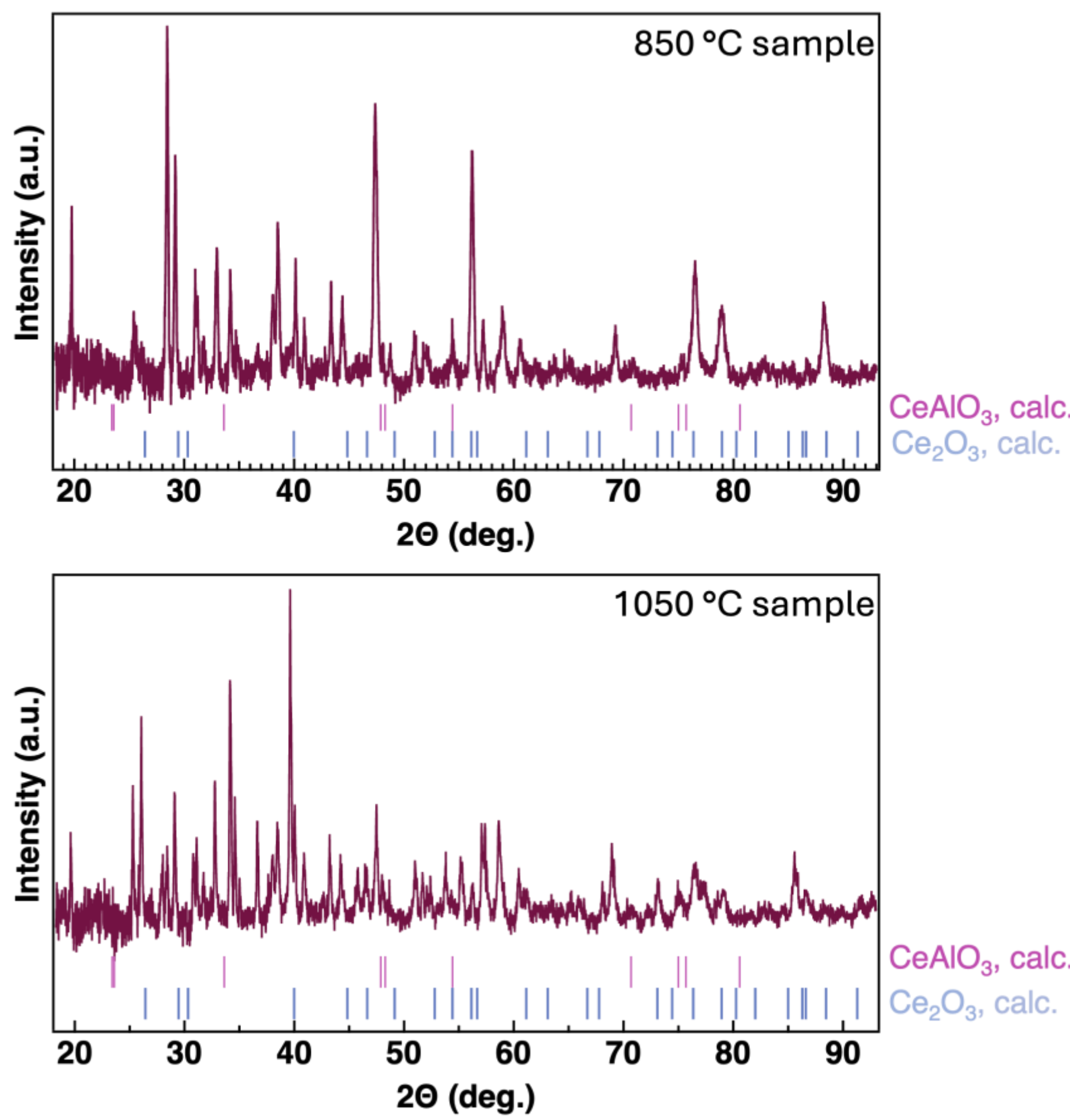

Figure S3. Powder patterns for samples heated to 850 °C (top) and 1050 °C (bottom) compared to $CeAlO_3$[5] (purple) and Ce_2O_3[6] (blue) to demonstrate absence of these compounds in the final products.

C. Infrared (IR) Spectroscopy

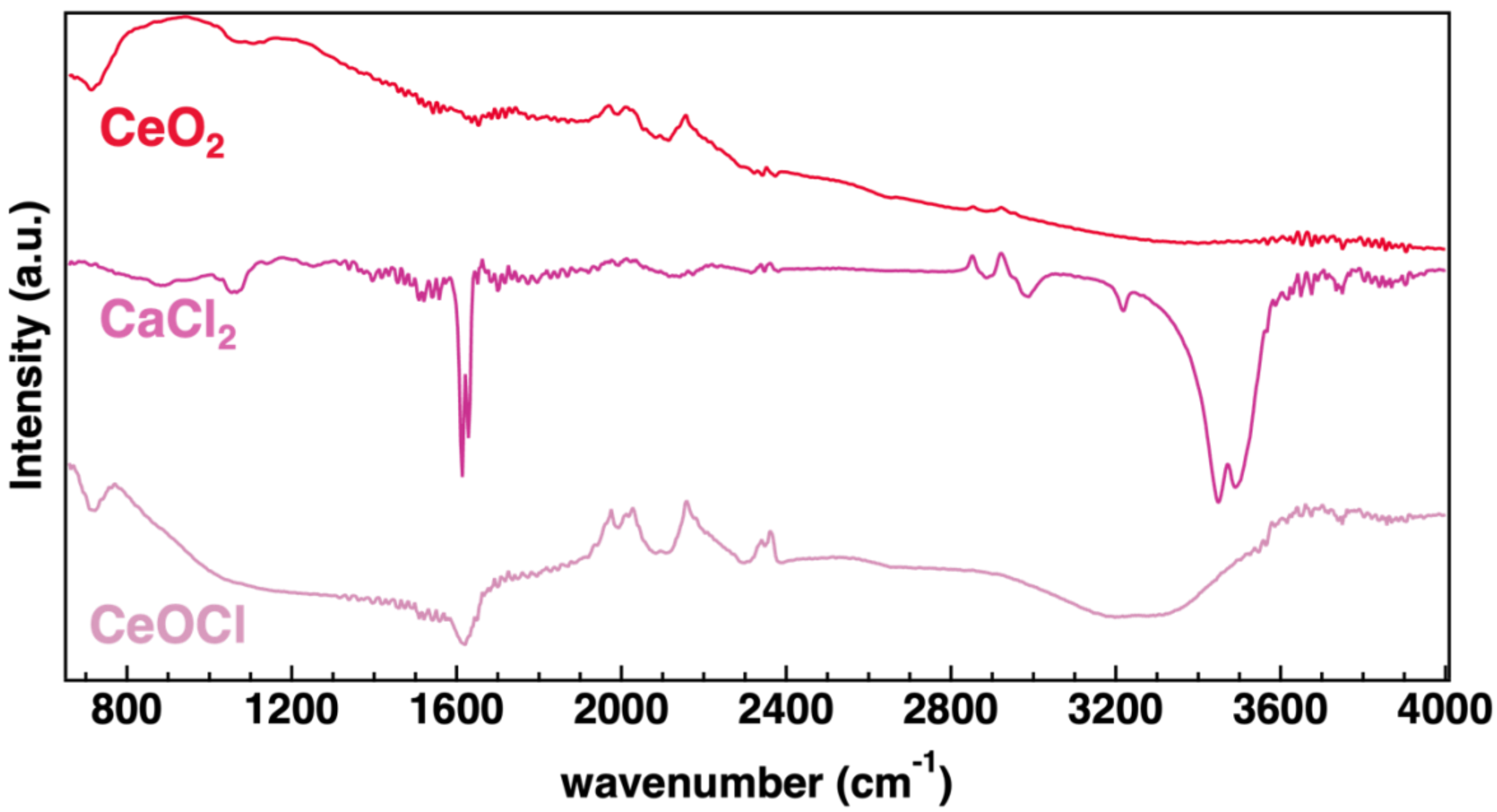

Figure S4. Reflectance IR of CeOCl (bottom, light pink), compared to $CaCl_2$ (middle, dark pink) and CeO_2 (top, red).

D. References

(1) Hölsä, J.; Lahtinen, M.; Lastusaari, M.; Valkonen, J.; Viljanen, J. Stability of Rare-Earth Oxychloride Phases: Bond Valence Study. *J. Solid State Chem.* **2002**, *165* (1), 48–55. https://doi.org/10.1006/jssc.2001.9491.

(2) Howard, C. J.; Kennedy, B. J.; Curfs, C. Temperature-Induced Structural Changes in $CaCl_2$, $CaBr_2$, and $CrCl_2$: A Synchrotron x-Ray Powder Diffraction Study. *Phys. Rev. B* **2005**, *72* (21), 214114. https://doi.org/10.1103/PhysRevB.72.214114.

(3) Yashima, M.; Kobayashi, S.; Yasui, T. Crystal Structure and the Structural Disorder of Ceria from 40 to 1497 °C. *Solid State Ion.* **2006**, *177* (3–4), 211–215. https://doi.org/10.1016/j.ssi.2005.10.033.

(4) Ishizawa, N.; Miyata, T.; Minato, I.; Marumo, F.; Iwai, S. A Structural Investigation of α-Al_2O_3 at 2170 K. *Acta Crystallogr. B* **1980**, *36* (2), 228–230. https://doi.org/10.1107/S0567740880002981.

(5) Tanaka, M.; Shishido, T.; Horiuchi, H.; Toyota, N.; Shindo, D.; Fukuda, T. Structure Studies of $CeAlO_3$. *J. Alloys Compd.* **1993**, *192*, 87–89.

(6) Bärnighausen, H.; Schiller, G. The Crytal Structure of A-Ce_2O_3. *J. Common Met.* **1985**, No. 110, 385–390.
